# Evaluation of particle size and feed form on performance, carcass characteristics, nutrient digestibility, and gastrointestinal tract development of broilers at 39 d of age

**DOI:** 10.1016/j.psj.2024.103437

**Published:** 2024-01-08

**Authors:** M.S. Rueda, S. Bonilla, C. de Souza, J.D. Starkey, C.W. Starkey, L. Mejia, W.J. Pacheco

**Affiliations:** ⁎ Department of Poultry Science, Auburn University, Auburn, AL 36830, USA; † Cobb Vantress, Siloam Springs, AR 72761, USA

**Keywords:** breast myopathy, GI tract, gizzard, reverse peristalsis

## Abstract

The objective of this study was to evaluate combined effects of corn particle size and feed form on performance, carcass characteristics, nutrient digestibility, and gastrointestinal tract development of broilers from 1 to 39 d of age. A total of 1,800 days old, male Cobb 500 broilers were randomly assigned to 9 dietary treatments with 8 replicate pens (25 birds/pen). The experiment consisted of a factorial arrangement of 3 corn particle sizes (750, 1,150, and 1,550 μm) and 3 feed forms (mash, 3- and 4-mm pellets) provided from 1 to 39 d. Titanium dioxide (**TiO_2_**) was added as an indigestible marker (0.5%) during the finisher phase (27–39 d) to determine nutrient digestibility. Feed intake (**FI**), body weight (**BW**), and feed conversion ratio (**FCR**) were determined at 17, 27, and 39 d of age, with FCR adjusted for mortality. On d 40, 10 birds/pen were randomly selected and processed for meat yield determination. Data were analyzed as a 3×3 factorial (particle size x feed form) arrangement of treatments. Broilers fed 3- and 4-mm pellets had increased (*P* < 0.05) BW, FI, and lower FCR than broilers fed mash diets at 39 d of age. At 39 d of age, broilers fed diets with 750 µm corn particle size had heavier (*P* < 0.05) BW and increased FI than broilers fed diets with corn particle sizes of 1,150 and 1,550 µm. At 39 d of age FCR was unaffected by corn particle size. Heavier (*P* < 0.05) carcass and breast weights were observed for broilers fed 3-mm pellets. Broilers fed diets with corn particle size of 750 µm had heavier (*P* < 0.05) carcass and breast weight than broilers fed diets with 1,550 µm. Digestibility of nutrients was higher (*P* < 0.05) in pelleted diets, particularly when corn particle size was increased from 750 to 1,550 µm. Breast myopathies such as wooden breast (**WB**) and spaghetti meat (**SM**), were greater (*P* < 0.05) in broilers fed 3-mm pellets compared to mash diets. In conclusion, broilers fed 3- and 4-mm pelleted diets had greater nutrient digestibility and improved broiler performance compared to broilers fed mash diets.

## INTRODUCTION

Cereal grains are typically ground prior to their incorporation into poultry diets to increase steam penetration during feed conditioning and particle agglomeration during pelleting (Reimer, 1992; Behnke, 2001). However, feed is rapidly dissolved in the crop after consumption, and fine particles limit gizzard development due to a lack of mechanical stimulation (Engberg et al., 2002; Svihus, 2011). Whole grains or coarse cereal particles stimulate gizzard development, reverse peristalsis, and holding capacity, resulting in reduced passage rate and improved nutrient digestibility (Svihus, 2011).

Reduced feed wastage, mealtime, selective feeding, and nutrient segregation are some benefits obtained by pelleting, as well as increments in feed consumption, Body weight gain (BWG), bird uniformity, resting time, and feed efficiency (Behnke, 1996; Amerah et al., 2007). However, additional grinding during pelleting, as feed is compressed between the pellet die and rolls, appears to influence gastrointestinal tract development. In a study by Nir et al. (1994) lower gizzard weights were reported in birds fed pellets compared to mash-fed birds. Variations in pellet diameter have also been evaluated to determine optimum pellet size on broiler performance (Cerrate et al., 2009; Sundu et al., 2009; Abdollahi et al., 2012; Rubio et al., 2020). The objective of this study was to evaluate the interactive effects of feed form, corn particle size, and pellet diameter on growth performance, digestive tract development, carcass yield, and nutrient digestibility of modern broiler strains from 1 to 39 d of age. We hypothesized that inclusion of coarse particles (>1,000 µm) in pelleted diets can increase gizzard development and nutrient digestibility regardless of the grinding effect that can occur during the pelleting process.

## MATERIALS AND METHODS

### Animal Care

This experiment was conducted at Auburn University Charles C. Miller, Jr. Poultry Research and Education Center. All procedures involving live birds were approved by Auburn University Institutional Animal Care and Use Committee (PRN 2019-3517).

### Bird Management

A total of 1,800-day-old male Cobb 500 broilers obtained from a commercial hatchery were weighed and randomly distributed among 72 floor pens (9 treatments with 8 replicate pens each) in an environmentally controlled house (25 birds/pen; 0.10m^2^/bird). Birds and feed were weighed per pen to determine body weight (**BW**), feed intake (**FI**), and feed conversion ratio (**FCR**) at 17, 27, and 39 d of age. A solid-sided house was used as grow-out facility, with negative-pressure ventilation system equipped with exhaust fans, forced-air heaters, cooling pads, and electronic controllers to manage temperature and ventilation. Feed and water were offered ad libitum with 1 tube feeder and 6 nipple drinkers in each pen. Chicks received 0.7 kg of starter feed from d 1 to 17, 1.45 kg of grower feed from d 17 to 28, and finisher feed thereafter. Lighting program consisted of 23L:1D from d 1 to 7, 21L:3D from d 8 to 21, and 16L:8D from d 22 to 39. Environmental temperature was maintained at 33°C on d 1 and was gradually reduced to 18.3°C by 39 d of age. Mortality and housing conditions were monitored and recorded daily.

### Diets and Experimental Design

**Figure 1:**
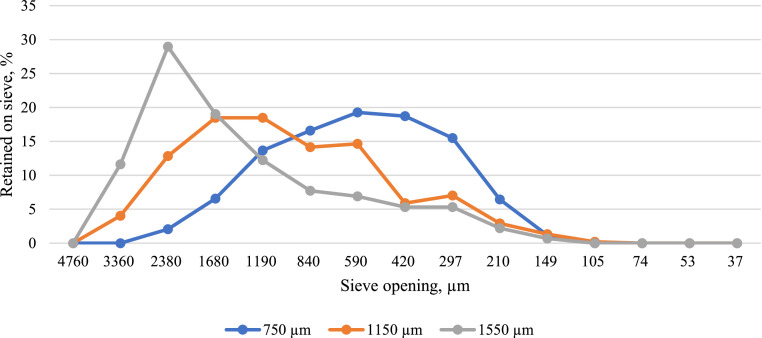
The geometric mean diameter by mass (D_gw_) and average particle size distribution (S_gw_) of corn before mixing was determined. Cobb x Cobb 500 male broilers were fed with diets containing 3 corn particle sizes. Dietary treatments (corn particle size: 750, 1,150, and 1,550 μm) were obtained by grinding whole corn in a hammermill equipped with a 7.94-mm screen and a variable frequency drive (VFD) on the main drive motor to modify hammer tip speed to achieve particle sizes of 701, 1,160, and 1,611 µm. The tip speed of the hammers was 9, 59, and 37 m/s to achieve particle sizes of 701, 1,160, and 1,611 µm.

Experimental diets (Table 1) were formulated to be identical in nutrient and ingredient composition in all treatments, differing only in corn particle size and feed form. Treatments consisted of 3 corn particle sizes (750, 1,150, and 1,550 μm) offered as mash, 3- and 4-mm pellets. Whole corn was ground with a hammermill (Model 11.5 and 38, Roskamp Champion, Waterloo, IA) equipped with 7.94-mm screen and a variable frequency drive on the main drive motor to adjust hammer tip speed and control geometric mean particle size by mass (D_gw_) to achieve the desired particle size (Figure 1). To obtain 750 µm corn particle size, the tip speed of the hammers was maintained at the maximum speed of 91 m/s and then reduced to 59 and 37 m/s to achieve the desired particle size of 1,150 and 1,550 µm. To ensure identical nutritional composition and particle size during the entire trial, total corn utilized for experimental diets was ground at the beginning of the experiment. After grinding corn, dry ingredients were blended for 150 s (30 s dry cycle and 120 s wet cycle) in a twin shaft mixer (Model 726, Scott Equipment Co., New Prague, MN) to produce mash diets. Pelleted diets were conditioned at 82°C for 45 s and pelleted either through a 3- or 4-mm pellet die (Model 1112-4, California Pellet Mill Co., Crawfordsville, IN). Pellets were then cooled with ambient air using a counter-flow pellet cooler (Model CC0909, California Pellet Mill Co.). Starter feed for pelleted treatments was crumbled in a crumbler with manual roll adjustment (Model 624SS, California Pellet Mill Co.). Titanium dioxide (**TiO_2_**) was added as an indigestible marker at 0.5% inclusion to determine nutrient digestibility during the finisher period (27–39 d of age).

**Table 1:** Ingredient and nutrient composition of dietary treatments varying in feed form and particle size fed to Cobb × Cobb 500 male broilers from 1 to 39 d of age.

Ingredients, % “as fed”	Starter	Grower	Finisher
Corn 7.4% CP	56.62	62.59	66.56
Soybean meal 46.9% CP	37.79	32.08	27.33
Corn oil	2.05	2.04	3.23
Dicalcium phosphate	1.19	0.85	0.88
Limestone	0.92	0.80	0.82
Titanium dioxide	0.00	0.00	0.50
Salt 96+%	0.37	0.37	0.38
DL methionine	0.36	0.33	0.29
L-Lysine HCl	0.25	0.21	0.22
Choline Cl-60%	0.06	0.06	0.07
L-Threonine 98%	0.16	0.12	0.08
Trace-mineral premix^1^	0.10	0.10	0.10
Vitamin premix^2^	0.10	0.08	0.05
Quantum phytase	0.01	0.01	0.01
Calculated analysis, % (unless otherwise noted)			
AME_n_ kcal/kg	2,975	3,065	3,110
Crude protein	22.16	19.88	17.95
Digestible lys	1.26	1.10	1.00
Digestible Thr	0.86	0.75	0.65
Digestible TSAA^3^	0.94	0.86	0.78
Calcium	0.90	0.76	0.76
Available phosphorus	0.45	0.38	0.38

The analyzed nutritional values of the finisher diet were 16.80%, 5.19%, and 4,035 kcal/kg for CP, crude fat, and gross energy, respectively.

1 Mineral premix include per kg of diet: Mn (manganese sulfate), 120 mg; Zn (zinc sulfate), 100 mg; Fe (iron sulfate monohydrate), 30 mg; Cu (tri-basic copper chloride), 8 mg; I (ethylenediamine dihydriodide), 1.4 mg; and Se (sodium selenite), 0.3 mg.

2 Vitamin premix includes per kg of diet: Vitamin A (Vitamin A acetate), 18,7390 IU; Vitamin D (cholecalciferol), 6,614 IU; Vitamin E (DL-alpha tocopherol acetate), 66 IU; menadione (menadione sodium bisulfate complex), 4 mg; Vitamin B12 (cyanocobalamin), 0.03 mg; folacin (folic acid), 2.6 mg: D-pantothenic acid (calcium pantothenate), 31 mg; riboflavin (riboflavin), 22 mg; niacin (niacinamide), 88 mg; thiamin (thiamin mononitrate), 5.5 mg; D-biotin (biotin), 0.18 mg; and pyridoxine (pyridoxine hydrochloride), 7.7 mg.

3 TSAA = Total sulfur amino acids.

### Measurements

Geometric mean diameter of particles by mass (D_gw_) for different treatments was determined according to the American Society of Agricultural and Biological Engineers (**ASABE**) method S319.4 (ASABE Standards, 2008) by collecting 3 samples of ground corn. A total of 5 feed bags per treatment with an average weight of 22.7 kg were collected during bagging at evenly spaced intervals and used as replicates to evaluate combined effects of corn particle size and pellet diameter on pellet durability index (**PDI**) of grower and finisher feeds. Pellet durability was determined according to procedures described in ASABE Standard S269.5 (ASABE Standards, 2012). Pellets of 3- and 4-mm diameter were sifted using a 2.8 mm (0.111 in) and a 3.4 mm (0.132 in) screen size, following recommendations for sieve size of various diameters of crumbles and animal feed, found in method S319.4 (ASABE Standards, 2008).

Birds and feed were weighed at 17, 27, and 39 d of age to determine FI, BW, and FCR. Mortality was removed, weighed, and recorded daily. Weight of mortality and days mortality lived were used to adjust FCR for each pen. On d 39, one bird per pen was selected within +/- 3% of pen mean weight, euthanized using CO_2_ followed by cervical dislocation, and gastrointestinal tract organs; gizzard, proventriculus, pancreas, duodenum, jejunum, and ileum were collected, emptied, and weighed to determine their relative weight. In addition, relative lengths of duodenum, jejunum, and ileum were measured with a flexible metric tape on a metal surface table to prevent inadvertent stretching. Lengths of duodenum (from the pyloric junction to the distal-most point of insertion of the duodenal mesentery), jejunum (from the distal-most point of insertion of the duodenal mesentery to the junction with Meckel's diverticulum), and ileum (from the junction with Meckel's diverticulum to the ileocecal junction) were measured and after separating each segment from any adherent mesentery their empty weight was determined.

Furthermore, on d 39, 4 birds per pen were randomly selected and euthanized using CO_2_ followed by cervical dislocation to collect ileal contents. Ileal digesta was collected (2 cm posterior of Meckel's diverticulum to 2 cm anterior of the ileal-cecal junction) by gently squeezing the ileal contents in a manner that provided enough sample (10–12 g after freeze-dried) for digestibility analysis. Ileal digesta samples were frozen, lyophilized, and ground. Experimental diets and ileal digesta were analyzed for crude protein (**CP**), crude fat, and gross energy (**GE**) content. Crude protein was analyzed via the Dumas method (method 990.03; AOAC International 2006) using a N analyzer (Rapid N Cube, Elementar Analysensyteme GmbH, Hanau, Germany) with CP being calculated by multiplying percent N by a correction factor (6.25). Crude fat concentration was estimated by boiling samples in hexane (method 2003.06; AOAC International, 2006) using a fat extractor (Soxtec model number 2043, Foss North America, Inc., Eden Prarie, MN). Gross energy was determined using an adiabatic bomb calorimeter (Parr, 6300, Parr Instruments, Moline, IL) standardized with benzoic acid. TiO_2_ concentration was determined following the procedures of Short et al. (1996). Apparent ileal digestibility (**AID**) of CP, crude fat, and energy were calculated using the following equation adapted from Stein et al. (2007):

$$\text{AID}\;\%=\left\{\left[{\left(\text{Nutrient}/\text{Ti}O_{2}\right)}_{\text{diet}}-\left(\text{Nutrient}/\text{Ti}O_{2}\right)\text{digesta}\right]/\left(\text{Nutrient}/\text{Ti}O_{2}\right)\text{diet}\right\}\;\text{and}\;100$$

were (nutrient/TiO_2_) = ratio of CP, crude fat or GE to TiO_2_ in diet or ileal digesta. Energy digestibility values obtained from this equation were multiplied by GE content of feed to calculate apparent ileal digestible energy (**IDE**) in units of kcal/kg (Gautier and Rochell, 2020). Titanium dioxide recovery on feed samples ranged from 0.49 to 0.58% and were similar to target concentration of 0.50%

On d 39, 10 birds per pen were randomly selected, wing banded, and marked with food-grade dye to evaluate carcass and parts weight and yield. Feed was removed from each pen 10 h prior to processing, but birds had access to drinking water during feed removal period. On d 40, selected birds were loaded into coops and transferred to Auburn University Pilot Processing Plant. Broilers were electrically stunned, exsanguinated, scalded, and mechanically eviscerated. After processing, carcasses were chilled in ice water for 4 h and then excess water was drained for approximately 5 min prior to determining chilled carcass weight. Carcass yield was calculated in relation to live BW, whereas parts’ yield was calculated as a percentage of chilled carcass weight. On d 41, carcasses were deboned using stationary cones to determine meat yield (breasts, wings, and tenders), severity, and incidence of breast myopathies. During deboning, breast fillets (*pectoralis major*) and tenders (*pectoralis minor*) were separated and weighed to determine yield. Wooden breast (**WB**) was evaluated by hand palpation, scoring breast fillets from normal (score 0), mild (score 1), moderate (score 2), and severe (score 3). Spaghetti meat (**SM**) was evaluated by pinching the top surface of breast fillets and looking for turgor (presence or absence).

### Statistical Analyses

Data were analyzed as a 3 × 3 factorial (feed form x corn particle size) arrangement of treatments. Pen location was the blocking factor. Pen was considered the experimental unit with 8 replicate pens per treatment. Data were analyzed using the MIXED procedure of JMP software (SAS Institute Inc., Cary, NC) with the following mixed-effects model:

$$Y_{ijk}=\mu +\rho_{i}+\tau_{j}+{\left(\rho \tau \right)}_{ij}+\delta_{k}+\varepsilon_{ijk}$$

where *Y_ijk_* = observed response of the bird in each pen; *μ* = is the overall mean; ρ*_i_* is the effect of the *i*th level of feed form, *τ_j_* is the effect of the *j*th level of corn particle size, (ρ*_τ_)_ij_* is the effect of the interaction between the *i*th level of feed form and the *j*th level of corn particle size, *δ_k_* is the effect of the *k*th block and ε*_ijk_* is the residual error when pen was regarded as an experimental unit, normally and independently distributed with mean 0 and variance σ^2^. Least squares means among the 9 treatments were compared using Tukey's HSD procedure with statistical significance considered at *P* ≤ 0.05 unless otherwise indicated.

Pellet durability data were analyzed as a 2 × 3 factorial of pellet diameter and corn particle size. Data were analyzed using the MIXED procedure of JMP software (SAS Institute Inc.) with the following mixed-effects model:

$$Y_{ijk}=\mu +\rho_{i}+\tau_{j}+{\left(\rho \tau \right)}_{ij}+\delta_{k}+\varepsilon_{ijk}$$

where *Y_ijk_* = observed response of pellet durability; *μ* = is the overall mean; ρ*_i_* is the effect of the *i*th level of pellet diameter, *τ_j_* is the effect of the *j*th level of corn particle size, (ρ*_τ_*)*_ij_* is the effect of the interaction between the *i*th level of pellet diameter and the *j*th level of corn particle size, *δ_k_* is the effect of the *k*th block and ε*_ijk_* is the residual error when each collected bag of feed was regarded as an experimental unit, normally and independently distributed with mean 0 and variance σ^2^. Least squares means among the 6 treatments were compared using Tukey's HSD procedure with statistical significance considered at *P* ≤ 0.05 unless otherwise indicated.

Incidence and severity of breast myopathies was analyzed using the GLIMMIX procedure of SAS (PC version 9.4, SAS Institute Inc.). Satterthwaite adjustment was used to correct degrees of freedom with pen serving as the experimental unit. Proportional data were analyzed using the events/trials syntax with a binomial distribution and both continuous and proportional data were analyzed using an R-side covariance structure. Data were analyzed as a 2-way ANOVA and least squares means were separated using the PDIFF option for multiple means comparisons and considered significantly different when *P* ≤ 0.05 unless otherwise indicated.

## RESULTS AND DISCUSSION

### Feed Quality Parameters

Geometric mean diameter by mass (D_gw_) and particle size distribution of ground corn used during the whole experiment are shown in Figure 1. Average particle size of corn for the 3 different treatments was 701, 1,160, and 1,611 µm and was close to estimated values. The effect of pellet diameter and corn particle size on pellet quality is presented in Table 2. Pellet durability was higher (*P* < 0.05) in grower diets with corn ground to 1,550 µm and pelleted with a 3-mm die, while lowest PDI in diets with corn particle size of 750 and 1,550 µm pelleted with a 4-mm die. Similarly, PDI was higher (*P* < 0.05) in finisher diets with corn particle size of 1,550 µm pelleted with a 3-mm die, and lower in diets with corn ground to 750 µm pelleted with a 4-mm die. In a study by Cerrate et al. (2009) higher pellet quality was reported in diets pelleted with a 1.59-mm die compared to a 3.17-mm die. According to Abdollahi et al. (2012, 2013a) and Löwe (2005), as pellet diameter decreases, heat transfer to the center of pellets is increased as well as starch gelatinization in the surface of pellets, resulting in more durable pellets. Furthermore, Rubio et al. (2017) reported linear increments in PDI as corn particle size increased from 629 to 1,779 µm, particularly when PDI was analyzed using the Holmen tester. The authors suggested that a mesh size of 1.58 mm, could have retained particles >1,580 µm when determining pellet durability. In contrast, several authors have reported enhanced pellet durability with reduced particle size (Wondra et al., 1995; Chewning et al., 2012; Abadi et al., 2019). Greater surface area for steam penetration and particle binding is achieved with reduced particle size, resulting in higher pellet durability (Reimer, 1992; Behnke, 2001; Abadi et al., 2019). Although sieve size was selected using ASABE standards, coarse particles of corn or other cereals can be retained on top of sieve and counted as whole pellets during PDI determination, leading to confounding and contrasting results among authors.

**Table 2:** Effects of pellet diameter and corn particle size on pellet durability index (PDI) of grower and finisher feeds.^1^^,^^2^

Parameters		Grower	Finisher
*Interaction effects*			
3-mm pellet	750	91.97^b^	86.62^b^
3-mm pellet	1,150	92.59^b^	87.99^b^
3-mm pellet	1,550	93.81^a^	89.72^a^
4-mm pellet	750	89.18^d^	77.94^e^
4-mm pellet	1,150	90.81^c^	80.18^d^
4-mm pellet	1,550	89.74^d^	84.89^c^
SEM^3^		0.24	0.35
*Main effects*			
3-mm pellet		92.79	88.11
4-mm pellet		89.91	81.00
SEM		0.16	0.25
	750	90.58	82.28
	1,150	91.70	84.08
	1,550	91.78	87.31
SEM		0.20	0.31
*P-values*			
Pellet diameter		<0.0001	<0.0001
Particle size		<0.0001	<0.0001
Pellet diameter × particle size		<0.0001	<0.0001

a-c Means were compared using Tukey's HSD procedure with statistical significance considered at *P* ≤ 0.05 unless otherwise indicated. Least squares means within a column with different superscripts differ significantly.

1 Values are least squares means of duplicates of 5 feed bags per treatment.

2 PDI: Pellet durability index was calculated by dividing weight of pellets after tumbling/weight of pellets before tumbling and multiply it by a 100.

3 SEM: Standard error of the mean for feed form and corn particle size interactions and main effects.

### Broiler Growth Performance

Interactions and main effects of feed form and corn particle size on performance variables at 27 and 39 d are presented in Table 3. An interaction (*P* < 0.05) between corn particle size and feed form was found on BW at 17 d and on FCR at 17 and 27 d. On d 17, BW of broilers fed mash diets decreased (*P* < 0.05) as corn particle size increased from 750 µm to 1,150 µm. Furthermore, FCR increased (*P* < 0.05) at 17 and 27 d of age as corn particle size was increased from 750 µm to 1,150 µm in birds fed mash diets. However, corn particle size did not influence BW and FCR at 17 and 27 d when broilers were fed 3- and 4-mm pellets. Similar interactions have been previously reported where broilers fed pelleted diets remain unaffected by varying particle size (Parsons et al., 2006; Amerah et al., 2007; Chewning et al., 2012; Lv et al., 2015; Naderinejad et al., 2016; Abadi et al., 2019). In addition, several authors have reported higher FCR as particle size increased, particularly in young chicks, likely due to higher energy expenditure for gizzard contraction and reduced feed intake (Killburn and Edwards, 2001; Parsons et al., 2006; Chewning et al., 2012; Xu et al., 2015b). However, Amerah et al. (2007) reported lower FCR when broilers were fed wheat-based diets of 1,164 µm compared to 839 µm and attributed the improvement in FCR to improved gastric function and peptide digestion as well as reduced passage rate, allowing longer exposure of digesta to digestive enzymes for better absorption of nutrients.

**Table 3:** Growth performance of Cobb x Cobb 500 male broilers fed diets varying in feed form and corn particle size from 1 to 39 d of age.^1^

Parameters		Body weight, g			Feed intake, g			Feed conversion ratio^2^, g:g		
		1–17 d	1–27 d	1–39 d	1–17 d	1–27 d	1–39 d	1–17 d	1–27 d	1–39 d
*Interaction effects*										
Mash	750 μm	631^b^	1,411	2,829	787	1,963	4,270	1.34^b^	1.47^b^	1.58
Mash	1,150 μm	595^bc^	1,344	2,754	750	1,909	4,184	1.37^ab^	1.51^a^	1.60
Mash	1,550 μm	564^c^	1,290	2,711	732	1,890	4,135	1.42^a^	1.55^a^	1.61
3-mm pellet	750 μm	795^a^	1,764	3,333	919	2,335	4,817	1.22^c^	1.38^c^	1.52
3-mm pellet	1,150 μm	758^a^	1,697	3,226	880	2,238	4,626	1.24^c^	1.39^c^	1.51
3-mm pellet	1,550 μm	766^a^	1,712	3,237	910	2,298	4,738	1.26^c^	1.41^c^	1.54
4-mm pellet	750 μm	749^a^	1,688	3,227	882	2,241	4,677	1.26^c^	1.39^c^	1.53
4-mm pellet	1,150 μm	755^a^	1,658	3,224	893	2,248	4,629	1.26^c^	1.42^c^	1.51
4-mm pellet	1,550 μm	753^a^	1,683	3,181	885	2,245	4,600	1.26^c^	1.40^c^	1.52
SEM^3^		10.8	25.6	42.6	13.5	31.2	48.0	0.1	0.1	0.1
*Main effects*										
Feed form										
Mash		596	1,348^b^	2,765^b^	756^b^	1,921^b^	4,196^b^	1.38	1.51	1.60^a^
3-mm pellet		773	1,724^a^	3,265^a^	903^a^	2,291^a^	4,727^a^	1.24	1.39	1.52^b^
4-mm pellet		752	1,676^a^	3,211^a^	886^a^	2,245^a^	4,635^a^	1.26	1.40	1.52^b^
SEM		7.19	14.9	24.2	8.1	18.0	27.7	0.01	0.01	0.01
Particle size, μm										
750		725	1,621^a^	3,130^a^	862	2,180	4,588^a^	1.27	1.41	1.54
1,150		703	1,566^b^	3,068^ab^	841	2,132	4,480^b^	1.29	1.44	1.54
1,550		694	1,562^b^	3,043^b^	842	2,145	4,491^b^	1.31	1.45	1.56
SEM		7.19	14.9	24.2	8.1	18.0	27.7	0.1	0.1	0.1
*P-values*										
Feed form		<0.0001	<0.0001	<0.0001	<0.0001	< 0.0001	<0.0001	<0.0001	<0.0001	<0.0001
Particle size		0.0080	0.0099	0.0437	0.1004	0.1618	0.0135	<0.0001	<0.0001	0.1710
Feed form × particle size		0.0409	0.2307	0.7481	0.0898	0.4055	0.4002	0.0118	0.0014	0.3885

a–c Means were compared using Tukey's HSD procedure with statistical significance considered at *P* ≤ 0.05 unless otherwise indicated. Least squares means within a column with different superscripts differ significantly.

1 Values are least squares means of 8 replicate pens with 25 birds at placement.

2 Feed conversion ratio, corrected for mortality.

3 SEM: Standard error of the mean for feed form and corn particle size interactions and main effects.

At 39 d of age, no interactions were observed between feed form and corn particle size, suggesting that young broilers are more sensitive to changes in particle size and feed form as they are not able to efficiently consume and digest diets containing high inclusion of coarse particles. Nevertheless, influence of particle size is less evident in pelleted diets due to grinding that occurs in the pellet mill as feed is extruded between rolls and pellet die (Bonilla, 2022).

During the whole grow-out period, broilers fed 3- and 4-mm pellets had higher (*P* < 0.05) BW and FI and lower FCR in comparison to broilers fed mash diets. Several authors have reported improved performance in broilers fed pelleted compared to mash diets (Engberg et al., 2002; Dozier et al., 2010; Hu et al., 2012; Naderinejad et al., 2016; Abdollahi et al., 2018; Rubio et al., 2019). According to Amerah et al. (2007) pelleting reduces feed wastage, decreases energy expenditure during feeding, and increases feed consumption and nutrient digestibility leading to improved performance of broilers.

On d 27 and 39, broilers fed diets with corn ground to 750 µm had heavier (*P* < 0.05) BW (725, 1,621, and 3,130 g vs. 694, 1,562, and 3,043 g, respectively) than broilers fed diets with corn particle size of 1,550 µm. In a study by Nir et al. (1994) increased BW was reported in birds fed diets with corn particle size of 897 µm compared to 525 and 2010 µm. However, Amerah et al. (2008), Jacobs et al. (2010), and Rubio et al. (2020) reported no effects of corn particle sizes ranging from 284 to 1,779 µm on broiler performance during first 21 d of life. In contrast, Nir et al. (1994) reported increased BW at 21 d, in broilers fed diets with corn ground to 1,132 and 2,028 µm compared to 627 µm. Xu et al. (2015a) reported increased BW at 28, 35, and 42 d in broilers fed diets with 25 and 50% coarse corn inclusions (particle sizes of 541 and 640 µm, respectively) compared to broilers fed diets with 0% coarse corn inclusion (particle size of 432 µm).

Furthermore, FI was unaffected by corn particle size for d 17 and 27. However, broilers fed diets with corn particle size of 750 µm at 39 d of age, consumed more feed (*P* < 0.05) by 108 and 97 g compared to broilers fed diets with corn particle sizes of 1,150 and 1,550 µm, respectively. Chewning et al. (2012) reported higher FI in broilers fed diets with particle size of 600 µm compared to 300 µm, from 21 to 44 d of age. Zang et al. (2009) reported reduced ADFI throughout the 42-d trial in broilers fed diets with particle size of 597 µm compared to 953 µm. In contrast, Jacobs et al. (2010) reported no effects of corn particle size (557–1,387 µm) on FI throughout the 21-d trial.

The FCR at 39 d of age was unaffected by corn particle size. Parsons et al. (2006) reported improved feed efficiency in broilers (21–42 d of age) fed corn-soybean meal mash diets, as particle size increased from 781 to 2,242 µm. Similarly, Pacheco et al. (2013) reported improved FCR at d 14, 35, and 49, as the particle size of SBM increased from 352 and 465 µm to 971 and 1,080 µm. Singh et al. (2014) reported a quadratic effect on feed per gain, values increased as inclusion levels of coarse corn reached 300 g/kg in diet with a particle size of 877 µm, thereafter feed per gain decreased with inclusions of 450 and 600 g/kg in diets with particle sizes of 987 and 1,172 µm, respectively.

Throughout literature, effects of particle size on broiler performance are inconsistent as classification of particle size is very subjective where a fine particle size can vary between 300 and 839 µm (Amerah et al., 2007; Chewning et al., 2012). In addition, pelleting also affects the extent at which broiler performance is influenced by particle size, as it reduces initial particle size in the microstructure of pellets (Bonilla, 2022).

### Nutrient Digestibility

Effects of feed form and corn particle size on nutrient digestibility from 28 to 39 d are presented in Table 4. Interactions between feed form and particle size were found for AID of CP, crude fat, and IDE. Broilers fed diets with corn ground to 1,550 µm and 3- and 4-mm pellets had higher (*P* < 0.05) CP digestibility compared to broilers fed mash diets regardless of the particle size of corn and broilers fed diets with corn ground to 750 µm and fed 3-mm pellets. In contrast to our results, Naderinejad et al. (2016) and Abdollahi et al. (2018) reported reduced coefficient of apparent ileal digestibility of N in pelleted diets compared to mash diets. However, according to Rakic (2012) proteins are known to easily respond to heat when water is present, thus changing their 3-dimensional structure and physical behavior. During the pelleting process, both heat and water, in the form of vapor, are present, therefore, it can be suggested, a certain degree of protein denaturation occurs. In addition, disruption of ingredients’ cell wall makes protein and digesta more accessible to bile acids and digestive enzymes (Saunders et al., 1969; Abdollahi et al. 2013b; Naderinejad et al., 2016), suggesting increased nutrient digestibility due to pelleting.

**Table 4:** Apparent ileal digestibility of nutrients (%) and ileal digestible energy (kcal/kg) determined in broilers fed diets varying in feed form and corn particle size during the finisher phase (28–39 d).^1^

Parameters		Apparent ileal digestibility, %		Ileal digestible energy^2^, kcal/kg
		Crude protein	Crude fat	
*Interaction effects*				
Mash	750 μm	77.90bcd	88.16^b^	2,958^bc^
Mash	1,150 μm	76.57^cd^	83.31^c^	2,841^c^
Mash	1,550 μm	77.61^cd^	81.33^c^	2,854^c^
3-mm pellet	750 μm	76.08^d^	94.48^a^	2,991^bc^
3-mm pellet	1,150 μm	81.97^ab^	95.38^a^	3,066^ab^
3-mm pellet	1,550 μm	83.71^a^	96.14^a^	3,183^a^
4-mm pellet	750 μm	80.52abc	94.11^a^	3,046^ab^
4-mm pellet	1,150 μm	82.60^a^	94.28^a^	3,087^ab^
4-mm pellet	1,550 μm	83.35^a^	95.55^a^	3,123^ab^
SEM^3^		0.89	0.90	35.90
*Main effects*				
Feed form				
Mash		77.36	84.27	2,884
3-mm pellet		80.59	95.33	3,080
4-mm pellet		82.17	94.65	3,086
SEM		0.59	0.61	22.9
Particle size				
750 μm		78.15	92.31	2,999
1,150 μm		80.39	90.99	2,998
1,550 μm		81.57	90.95	3,053
SEM		0.60	0.62	23.40
*P-values*				
Feed form		<0.0001	<0.0001	<0.0001
Particle size		<0.0001	0.1701	0.1142
Feed form × particle size		0.0003	<0.0001	0.0027

a-d Means were compared using Tukey's HSD procedure with statistical significance considered at *P* ≤ 0.05 unless otherwise indicated. Least squares means within a column with different superscripts differ significantly.

1 Values are least squares means of duplicates of ileal contents of 4 birds per pen.

2 Ileal digestible energy, calculated by multiplying the AID of GE and GE of diet.

3 SEM: Standard error of the mean for feed form and corn particle size interactions and main effects.

Broilers fed 3- and 4-mm pellets had higher (*P* < 0.05) AID of fat compared to broilers fed mash diets. Corn particle size did not influence AID of fat in broilers fed 3- and 4-mm pellets, however in broilers fed mash diets fat digestibility was reduced from 88.16 to 83.31 and 81.33% as corn particle size increased from 750 to 1,150 and 1,550 µm, respectively. Similarly, Abdollahi et al. (2013b) reported improved coefficient of AID of fat by feeding pellets compared to mash diets. Bozkurt et al. (2019) reported increased ether extract digestibility in egg-laying pullets fed crumbles compared to mash diets, evidenced by an increment in pancreatic lipase activity. The higher fat digestibility observed in pelleted diets compared to mash diets could be due to the disruption of oil cell walls during the pelleting process leaving fat more available for digestion.

Ileal digestible energy was unaffected by corn particle size in broilers fed mash and 4-mm pelleted diets. However, in birds fed 3-mm pellets, IDE was higher (*P* < 0.05) by 192 kcal/kg when corn was ground to 1,550 µm compared to corn ground to 750 µm. Several authors have reported increased energy (Mtei et al., 2019) and starch digestibility (Ruhnke et al., 2015; Cordova-Noboa et al., 2020) with coarse particles in both mash and pelleted diets. Coarse particles stimulate gizzard function and its grinding capacity, increasing the surface area of digesta entering the small intestine and resulting in increased surface area for nutrient digestion and absorption (Svihus, 2011). Incremental improvements observed in IDE as corn particle size was increased are likely due to improved gastric activity as a result of increased gizzard grinding activity, lower passage rate, and better enzyme substrate interactions for nutrient digestion and absorption.

### Digestive Tract Development

Data for gastrointestinal tract development at 39 d of age is presented in Table 5. There were no significant interactions between corn particle size and feed form on gastrointestinal tract development at 39 d of age; therefore, only main effects will be discussed. Broilers fed mash diets exhibited greater (*P* < 0.05) relative weight of gizzard, pancreas, duodenum, jejunum, and ileum and greater (*P* < 0.05) relative length of duodenum compared to broilers fed pelleted diets. Similarly, other authors have reported greater relative gizzard weight in birds fed mash diets compared to birds fed pellets or crumbles (Hu et al., 2012; Lv et al., 2015; Abadi et al., 2019). In contrast, Dahlke et al. (2003) reported increased gizzard weights in birds fed pelleted diets. Resulting greater relative weights of gastrointestinal tract organs and intestine segments in broilers fed mash diets in this study can be due to 2 reasons. First, pelleting involves additional grinding, likely reducing initial particle size of preconditioned meal leading to reduced gizzard stimulation and development. According to Svihus (2011) when pellets are consumed, these are rapidly dissolved in the crop, exposing its microstructure. Pellets themselves (macrostructure) will usually not influence gizzard development or have other nutritional effects apart from increasing FI and reducing feed wastage (Engberg et al., 2002; Zaefarian et al., 2016). If pellet microstructure does not contain coarse particles, minimal gizzard stimulation will occur, and the gizzard will become a passage structure rather than a grinding organ. Second, relative weight is the ratio of the organ/segment weight to the broiler's BW, broilers fed mash diets resulted in lower BW increasing the relative weight of organs and intestine segments compared to broilers fed pelleted diets.

**Table 5:** Effect of feed form and corn particle size on digestive tract development of broilers from 1 to 39 d of age.^1^

Parameters		Relative weight, g/kg of BW						Relative length, cm/kg of BW		
		Gizzard	Proventriculus	Pancreas	Duodenum	Jejunum	Ileum	Duodenum	Jejunum	Ileum
*Interaction effects*										
Mash	750 μm	12.9	2.5	1.8	11.3	27.9	27.9	5.7	10.2	8.7
Mash	1,150 μm	14.0	2.8	2.0	11.7	28.8	29.7	6.4	10.8	9.2
Mash	1,550 μm	14.3	2.7	1.9	11.9	29.9	30.4	6.4	11.3	9.8
3-mm pellet	750 μm	8.1	2.5	1.6	9.4	24.4	24.0	5.6	11.0	8.3
3-mm pellet	1,150 μm	10.2	2.4	1.8	9.7	24.9	25.3	5.4	10.2	8.9
3-mm pellet	1,550 μm	10.6	2.5	1.7	9.6	24.1	25.1	5.9	10.5	9.1
4-mm pellet	750 μm	10.3	2.7	1.7	10.1	26.3	25.1	5.7	10.8	8.3
4-mm pellet	1,150 μm	10.2	2.5	1.7	9.9	25.1	25.4	5.4	10.4	8.2
4-mm pellet	1,550 μm	10.6	2.3	1.6	10.2	25.3	25.4	5.4	9.9	8.6
SEM^2^		0.5	0.1	0.1	0.3	0.7	1.0	0.2	0.4	0.4
*Main effects*										
Feed form										
Mash		13.7^a^	2.7	1.9^a^	11.7^a^	28.9^a^	29.4^a^	6.2^a^	10.8	9.2
3-mm pellet		9.6^b^	2.5	1.7^b^	9.6^b^	24.5^b^	24.8^b^	5.6^b^	10.6	8.8
4-mm pellet		10.4^b^	2.5	1.7^b^	10.1^b^	25.6^b^	25.3^b^	5.5^b^	10.4	8.4
SEM		0.3	0.1	0.1	0.2	0.4	0.6	0.2	0.1	0.2
Particle size, μm										
750		10.4^b^	2.6	1.7	10.3	26.2	25.7	5.6	10.7	8.4
1,150		11.5^ab^	2.6	1.8	10.5	26.3	26.8	5.7	10.5	8.7
1,550		11.8^a^	2.6	1.8	10.6	26.4	26.9	5.9	10.6	9.1
SEM		0.3	0.1	0.1	0.2	0.4	0.6	0.2	0.1	0.2
*P-values*										
Feed form		<0.0001	0.0957	0.0024	<0.0001	<0.0001	<0.0001	0.0030	0.5260	0.0597
Particle size		0.0053	0.8592	0.2372	0.5104	0.8998	0.2430	0.3635	0.8501	0.1135
Feed form × particle size		0.2245	0.0534	0.7661	0.8487	0.2366	0.8739	0.1353	0.1545	0.8158

a-b Means were compared using Tukey's HSD procedure with statistical significance considered at *P* ≤ 0.05 unless otherwise indicated. Least squares means within a column with different superscripts differ significantly.

1 Values are least squares means of 8 birds per treatment (1 bird/pen +/-3% of pen mean weight).

2 SEM: Standard error of the mean for feed form and corn particle size interactions and main effects.

Greater relative pancreas weight might indicate increased pancreatic activity. Engberg et al. (2002) reported higher activity of amylase, lipase, chymotrypsin, and trypsin of birds fed mash diets compared to pelleted diets. Additionally, reported a positive correlation between gizzard and pancreas relative weight, which may indicate that gizzard development and stimulation results in higher pancreatic activity.

Regarding intestinal segment lengths, several authors have reported reduced relative length of the intestine in birds fed pelleted diets compared to birds fed mash diets (Choi et al., 1986; Nir et al., 1994; Amerah et al., 2007; Naderinejad et al., 2016). In a study by Naderinejad et al. (2016), relative weights of intestinal segments were not influenced by feed form, but morphometry (villus height and crypt depth) in duodenum and jejunum was higher in birds fed pelleted diets than birds fed mash diets. Higher villus height, crypt depth and villus-crypt ratio are indicators of lower turnover rate of intestinal mucosa and lower maintenance requirements that results in higher growth rate and efficiency of the animal (Zang et al., 2009). In this study, morphometry was not evaluated but it resonates with literature that feeding pelleted feed to broilers results in improved growth rate and efficiency.

Regarding corn particle size, broilers fed diets with corn ground to 1,550 µm had greater (*P* < 0.05) gizzard relative weight than broilers fed diets with corn particle size of 750 µm. These results agree with other authors who have reported greater gizzard relative weight when coarse particles were included in broiler diets (Engberg et al., 2002; Parsons et al., 2006; Pacheco et al., 2013; Singh et al., 2014). Parsons et al. (2006) reported greater gizzard weight in broilers fed coarse corn mash (2,242 µm) compared to large, medium, and fine corn mash treatments with particle sizes ranging from 781 to 1,109 µm. In addition, Pacheco et al. (2013) reported greater gizzard relative weight in birds fed coarse corn (1,330 µm) compared to fine corn (520 µm). From these results it can be shown that gizzard relative weight is increased when coarse particles are included in broiler diets and stimulate gizzard development.

### Processing Weight and Yield

Data for carcass characteristics are presented in Table 6. There were no significant interactions between feed form and corn particle size on carcass characteristics; therefore, only main effects will be discussed. Broilers fed 3-mm pellets had higher (*P* < 0.05) cold carcass weight compared to broilers fed mash and 4-mm pellets (2,562 vs. 2,206 and 2,509 g). Carcass yield of broilers fed 3-mm pellets was greater (*P* < 0.05) by 2.69% than broilers fed mash diets. Broilers fed 3-mm pellets had higher (*P* < 0.05) breast weight compared to broilers fed mash and 4-mm pellets (683 vs. 564 and 663 g). Furthermore, breast meat yield of broilers fed mash diets was lower (*P* < 0.05) by 4.13 and 3.41% compared to broilers fed 3- and 4-mm pellets, respectively. Tender and wing weights were greater (*P* < 0.05) in broilers fed 3- and 4-mm pellets compared to broilers fed mash diets. However, wing yield was higher (*P* < 0.05) in broilers fed mash diets by 2.88 and 1.92% compared to broilers fed 3- and 4-mm pellets. Similarly, Rubio et al. (2019) reported greater carcass, breast and tender weight in broilers fed 3.3-mm pellets compared to broilers fed mash diets. Improvements in cold carcass, breast, tender, and wing weight were observed in birds receiving pelleted treatments, likely due to higher FI (531 g) and increased CP digestibility (4.81%) compared to birds fed mash diets. Higher feed consumption and nutrient digestibility in broilers fed pelleted diets permitted increased utilization of protein and energy to muscle growth compared to broilers fed mash diets evidenced in final BW, 2,765 g, mash-fed broilers compared to 3,265 and 3,211 g when broilers were fed 3- and 4-mm pellets.

**Table 6:** Processing characteristics of Cobb × Cobb 500 male broilers fed diets varying in feed form and corn particle size from 1 to 39 d of age.^1^

Parameters		Cold carcass		Breast		Tenders		Wings	
		(g)^1^	%^2^	(g)	%	(g)	%	(g)	%
*Interaction effects*									
Mash	750 μm	2,222	71.0	579	26.0	123	5.5	227	10.3
Mash	1,150 μm	2,224	73.6	568	25.5	117	5.3	232	10.5
Mash	1,550 μm	2,172	71.9	547	25.1	114	5.2	225	10.4
3-mm pellet	750 μm	2,617	72.5	696	26.6	140	5.3	262	10.0
3-mm pellet	1,150 μm	2,539	74.9	676	26.7	134	5.3	257	10.2
3-mm pellet	1,550 μm	2,539	75.0	677	26.6	134	5.3	257	10.1
4-mm pellet	750 μm	2,522	74.3	667	26.4	136	5.4	258	10.2
4-mm pellet	1,150 μm	2,525	74.9	665	26.4	133	5.3	256	10.2
4-mm pellet	1,550 μm	2,482	72.1	658	26.5	132	5.4	253	10.2
SEM^3^		22.3	0.8	8.3	0.2	1.7	0.1	2.4	0.1
*Main effects*									
Feed form									
Mash		2,206^c^	72.2^b^	564^c^	25.5^b^	118^b^	5.4	228^b^	10.4^a^
3-mm pellet		2,562^a^	74.2^a^	683^a^	26.6^a^	135^a^	5.3	258^a^	10.1^b^
4-mm pellet		2,509^b^	73.8^ab^	663^b^	26.4^a^	134^a^	5.4	255^a^	10.2^b^
SEM		12.9	0.5	4.8	0.1	0.9	0.1	1.4	0.1
Particle size, μm									
750		2,453^a^	72.6^b^	647^a^	26.4	133^a^	5.4^a^	249	10.2
1,150		2,426^ab^	74.5^a^	636^ab^	26.2	128^b^	5.3^b^	248	10.3
1,550		2,397^b^	73.0^ab^	627^b^	26.1	126^b^	5.3^b^	245	10.2
SEM		12.9	0.5	4.8	0.1	0.9	0.1	1.4	0.1
*P-values*									
Feed form		<0.0001	0.0113	<0.0001	<0.0001	<0.0001	0.4297	<0.0001	<0.0001
Particle size		0.0100	0.0156	0.0135	0.3019	<0.0001	0.0023	0.0932	0.1875
Feed form × particle size		0.2454	0.0693	0.5105	0.2046	0.6318	0.0970	0.2551	0.2599

a–c Means were compared using Tukey's HSD procedure with statistical significance considered at *P* ≤ 0.05 unless otherwise indicated. Least squares means within a column with different superscripts differ significantly.

1 Carcass weight was the average of 10 birds/pen randomly selected at d 39.

2 Percent of live weight was calculated by dividing part weight/live BW and 100.

3 SEM: Standard error of the mean for feed form and corn particle size interactions and main effects.

Corn particle size influenced (*P* < 0.05) most carcass characteristics except breast yield and wing weight and yield. Carcass weight of broilers fed diets with corn ground to 750 µm was heavier (*P* < 0.05) by 56 g than broilers fed diets with corn particle size of 1,550 µm, but similar to broilers fed diets with 1,150 µm corn particle size (2,453 g vs. 2,426 g). However, carcass yield of broilers fed diets with corn particle size of 1,150 µm was greater (*P* < 0.05) by 2.55% than broilers fed diets with corn particle size of 750 µm. Breast meat weight of broilers fed diets with corn ground to 750 μm were heavier (*P* < 0.05) by 20 g from broilers fed diets with corn particle size of 1,550 μm. Tender weight and yield of broilers fed diets with corn particle size of 750 μm were greater (*P* < 0.05) by 5 to 7 g and 1.85% from broilers fed diets with corn particle sizes of 1,150 and 1,550 μm, respectively. In a study by Lv et al. (2015), no effects of particle size (from 490 to 1,183 µm) on carcass characteristics were reported. However, Parsons et al. (2006) reported lower breast weight and yield as feed particle size was increased from 950 to 2,242 μm. Authors suggested feeding birds with coarse particles (2,242 μm) increases the proportion of feed energy directed to gizzard development and maintenance rather than to breast growth (Parsons et al., 2006).

### Breast Myopathies

**Figure 2:**
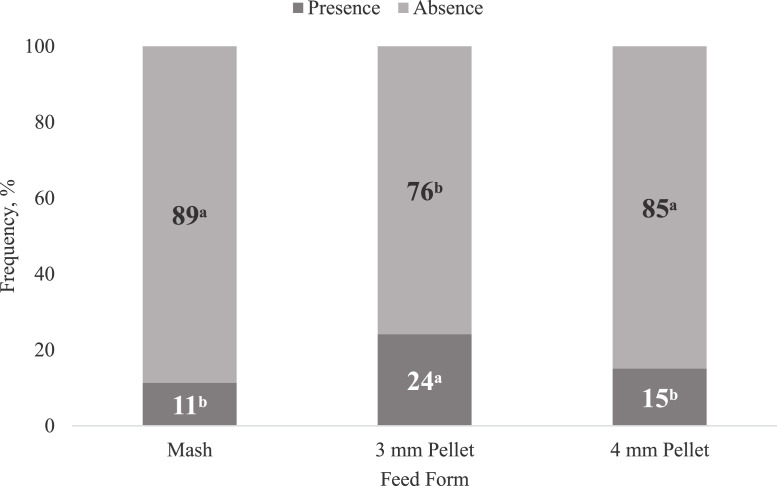
Effect of feed form on frequency (%) of spaghetti meat (SM) on Cobb × Cobb 500 male broilers fed diets varying in feed form and corn particle size from 1 to 39 d of age, n = 720. Least square means without a common letter are significantly different (*P* < 0.05). Values are presented as the mean percentage calculated from the incidence within each pen. Scores are based on the presence or absence of turgor on the top surface of breast fillets. *P*-value and SEM for SM are 0.0007 and 0.02578, respectively.

**Figure 3:**
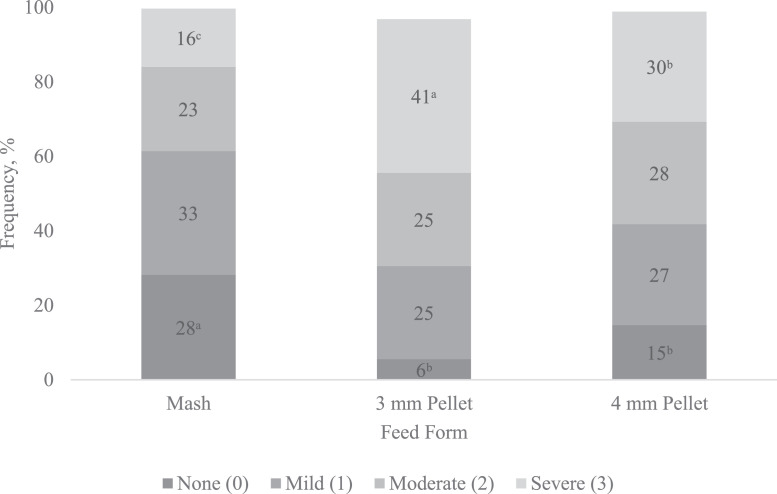
Effect of feed form on frequency and severity (%) of wooden breast on Cobb × Cobb 500 male broilers fed diets varying in feed form and corn particle size from 1 to 39 d of age, n = 720. Least square means without a common letter are significantly different (*P* < 0.05). Values are presented as the mean percentage calculated from the incidence within each pen. Scores are based on a 4-point scale (3 = severe, 2 = moderate, 1 = mild, 0 = normal). *P*-values for WB scores 0, 1, 2, and 3 are 0.0006, 0.2107, 0.5733, and <0.0001, respectively and SEM for WB scores 0, 1, 2, and 3 are 0.03864, 0.03518, 0.03371, and 0.03753, respectively.

Effects of feed form on the frequency of breast myopathies such as wooden breast and spaghetti meat are presented in Figure 2, Figure 3. Breast myopathies frequency were unaffected (*P* > 0.05) by corn particle size. However, broilers consuming mash and 4-mm pellets had 13 and 9 points, respectively, lower (*P* < 0.05) incidence of spaghetti meat in comparison to broilers fed 3-mm pellets (Figure 2). The higher incidence of spaghetti meat when broilers were fed 3-mm pellets could be due to higher breast weight (683 vs. 564 and 663 g; *P* < 0.05) of these broilers compared to broilers fed mash or 4-mm pellets.

Spaghetti meat is characterized by a separation of the bundles of muscle fibers mainly in cranial region of breast muscle leading to friability and loosening of muscle tissue (Baldi et al., 2018). Exudate and viscous material, as well as progressive rarefaction of endo- and perimysial connective tissue can be considered distinctive microscopic features of spaghetti meat defect (Baldi et al., 2018; Petracci et al., 2019; Bailey et al., 2020). Spaghetti meat is mainly characterized by a decrease in protein content and increase in fat and moisture level of chicken breast (Baldi et al., 2018). As with other myopathies, severity of spaghetti meat is variable, ranging from only a small part of breast muscle being affected, to the whole muscle showing the condition. In a study by Bailey et al. (2020), heritability estimate (0.074) for spaghetti meat was very low, indicating a strong influence of nongenetic factors for variation in expression of this characteristic. According to these authors, environmental and management factors (brooding, nutrition, temperature, and ventilation among others) have a major influence in muscle growth and development, which rely on satellite cells, multipotent stem cells that provide muscle fiber growth, repair, and maintenance (Moss and Leblond, 1970; Bailey et al., 2020). Consequences of negatively affected satellite cell function and activity can lead to decreased final breast yield and myopathies present in the composition of breast muscle (Bailey et al. 2020).

Severe WB frequency was as low as 16% in broilers fed mash diets compared to (*P* < 0.05) 41 and 30% in broilers fed 3- and 4-mm pellets, respectively. In addition, birds fed mash diets had 28% frequency of wooden breast score 0 compared (*P* < 0.05) to 6 and 15% in broilers fed 3- and 4-mm pellets respectively. According to Griffin et al. (2018), broilers with high growth rate, feed efficiency, and breast yield are more likely to develop breast myopathies, supporting our findings, where broilers fed 3-mm pellets had the heaviest breast weights differing from broilers fed mash diets. These abnormalities have been associated with rapid growth rate and muscle size affecting structure, metabolism, and repair mechanisms from satellite cells in breast muscles (Velleman et al., 2014; Velleman, 2015; Barbut, 2019).

## DISCLOSURES

The authors declare that the research was conducted in the absence of any commercial or financial relationships that could be construed as a potential conflict of interest.
